# Scapular Angiomatoid Fibrous Histiocytoma with EWSR1-CREB1 Fusion in an Adult Patient

**DOI:** 10.1155/2021/9434222

**Published:** 2021-09-30

**Authors:** Hiroshi Kobayashi, Naohiro Makise, Aya Shinozaki-Ushiku, Yuki Ishibashi, Masachika Ikegami, Shinji Kohsaka, Tetsuo Ushiku, Katsutoshi Oda, Kiyoshi Miyagawa, Hiroyuki Aburatani, Hiroyuki Mano, Sakae Tanaka

**Affiliations:** ^1^Department of Orthopaedic Surgery, Graduate School of Medicine, The University of Tokyo, Tokyo, Japan; ^2^Department of Pathology, Graduate School of Medicine, The University of Tokyo, Tokyo, Japan; ^3^Division of Cellular Signaling, National Cancer Center Research Institute, Tokyo, Japan; ^4^Division of Integrative Genomics, The University of Tokyo, Tokyo, Japan; ^5^Laboratory of Molecular Radiology, Center for Disease Biology and Integrative Medicine, The University of Tokyo, Tokyo, Japan; ^6^Genome Science Division, Research Center for Advanced Science and Technology, The University of Tokyo, Tokyo, Japan

## Abstract

**Background:**

Angiomatoid fibrous histiocytoma (AFH) is a rare intermediate malignant tumor that arises mainly in soft tissues, especially in the superficial extremities of patients younger than 30 years. There have been a few reports of AFH arising from sites other than soft tissue, including bone, and unusual site and age make it difficult to diagnose this rare tumor. *Case Presentation*. Here, we present a case of a 54-year-old man who was examined for chest pain, and computed tomography (CT) incidentally detected a bone tumor at the scapula with destruction of cortical bone and invasion into soft tissue. Magnetic resonance imaging revealed multiple cystic components with fluid-fluid levels. FDG-PET showed uptake at the axillary lymph node. The CT-guided needle biopsy revealed spindle cell sarcoma on histopathology. After neoadjuvant chemotherapy, a scapulectomy was performed. The final postresection histopathological diagnosis was the same as the preoperative diagnosis, and no obvious chemotherapeutic effect was observed. Next-generation sequencing of RNA isolated from paraffin-embedded tumor tissue revealed that these lesions harbored the *EWSR1-CREB1* fusion gene, and the tumor was diagnosed as AFH. C-reactive protein level, which was elevated preoperatively, decreased after the operation, and there was no recurrence or metastasis 5 years after the treatment.

**Conclusion:**

The diagnosis of AFH is difficult when the tumor has an atypical presentation. Comprehensive genomic analysis, especially RNA sequencing, is efficient in diagnosing this rare tumor. Moreover, magnetic resonance imaging findings identical to AFH in soft tissue, the presence of paraneoplastic symptoms such as elevated inflammatory markers, and lymph node swelling were clues towards suspecting this tumor.

## 1. Introduction

Angiomatoid fibrous histiocytoma (AFH) is a rare tumor of uncertain differentiation and is classified as intermediate (rarely metastasizing) [1]. Patients with AFH experience local recurrence in 15% and metastasis in 2%–5% cases [1]. It is mostly found in the subcutis of the extremities of patients younger than 30 years [1]. However, it has been reported that AFH can occur at other sites, including the neck, trunk, brain, lungs, peritoneum, gynecological tract, and bone [2, 3]. Furthermore, AFH could affect a broad range of ages, from infancy to as old as 85 years [4–6]. Patients mostly present with a slow-growing mass and rarely experience paraneoplastic symptoms such as weight loss, anemia, fever, night sweats, nausea, and vomiting [7].

In general, diagnosis of some bone and soft tissue tumors is difficult because of the rarity and lack of definite histopathological features. Recent advancements in comprehensive genetic analysis have shown that fusion genes can be used as diagnostic markers for bone and soft tissue tumors [1]. AFH has characteristic fusion genes, a member of the FET family, EWSR1 and FUS, and a member of the CREB-ATF basic leucine zipper family of transcription factors, ATF1 and CREB1 [8, 9].

Imaging features of AFH have been documented in a few studies, and AFH has well-circumscribed polycystic lesions with fluid-fluid levels [10, 11]. However, these reports were based on soft tissue AFH cases, and whether AFH at other parts, except for soft tissue, has the same imaging characteristics is unknown.

Herein, we describe a case of AFH arising from the scapula of a 54-year-old man. In this case, we describe the successful use of comprehensive genetic testing with a RNA panel to diagnose this rare tumor. In addition, AFH arising from the bone had imaging features identical to those of AFH in soft tissues.

## 2. Case Presentation

A 54-year-old man was suspected to have a bone tumor of the scapula when a computed tomography (CT) done to analyze the cause of chest pain incidentally revealed a lesion. The Institutional Review Board of the University of Tokyo approved this study, and written informed consent was obtained from the patient.

**Figure 1:**
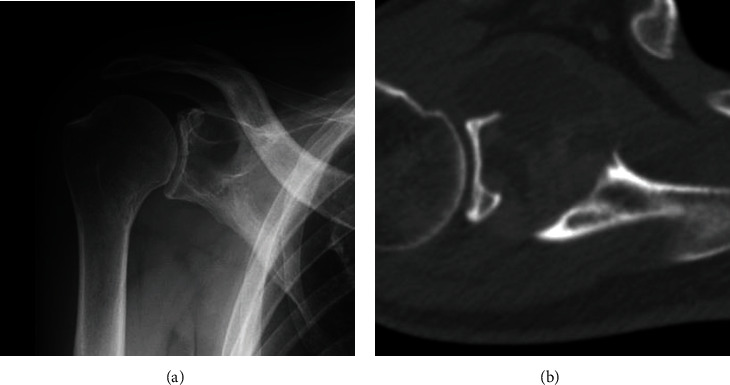
(a) Conventional radiograph of the right shoulder showing a lytic lesion involving the right scapula near the glenoid. (b) Computed tomography depicting the osteolytic lesion at the scapula with associated cortical destruction and extension into the surrounding soft tissue.

**Figure 2:**
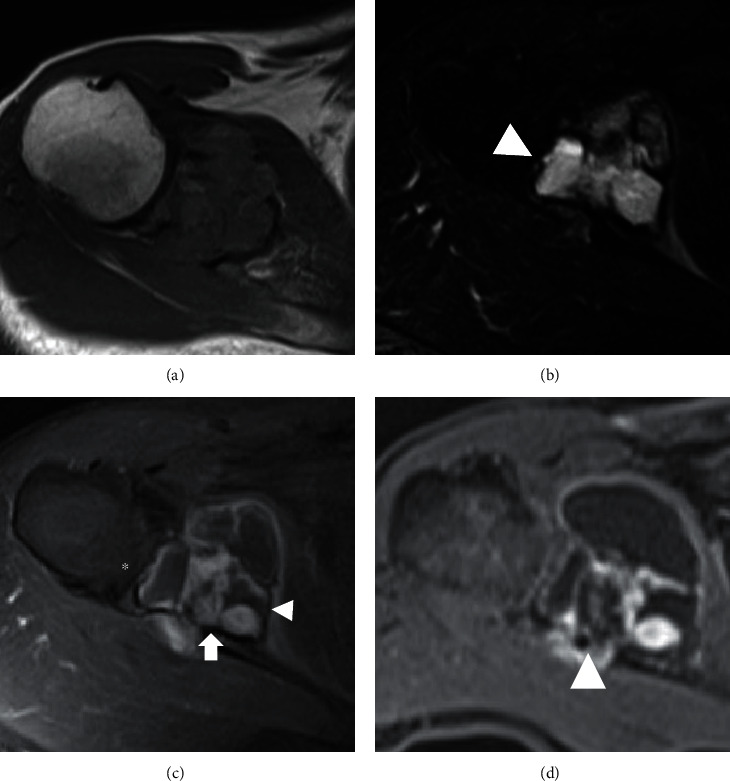
Magnetic resonance imaging at first presentation (a)–(c), and postchemotherapy (d). The lobulated tumor is located at the scapula and is seen invading into the anterior and posterior soft tissue. (a) Axial T1-weighted image shows slightly high signal intensity. (b) Axial T2-weighted image depicts high signal intensity-multilocular mass with low signal intensity rim, and a part of the nodule shows fluid-fluid level (white arrowhead). (c) Axial Gd enhanced T1-weighted image represents thin enhancement surrounding low-intensity rim and enhanced solid nodule (arrow). Partial invasion into soft tissue is observed (asterisk). (d) Axial Gd enhanced T1-weighted image after chemotherapy shows cystic change of solid nodule compared with prechemotherapy image in (c) panel.

**Figure 3:**
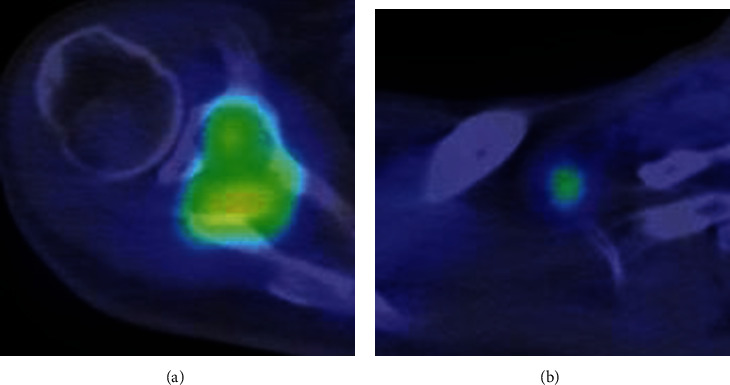
^18^F-FDG-PET/CT scan at first presentation. PET/CT fusion image shows increased FDG uptake in (a) the scapular tumor (SUV max 4.16) and (b) axillary lymph node (SUV max 2.8).

He had no pain around his right shoulder, and a blood test revealed an elevated C-reactive protein level of 7.69 mg/dl. Radiography of his right shoulder revealed a lytic lesion adjacent to the glenoid cavity (Figure 1(a)). CT revealed an osteolytic lesion destroying the cortical bone, and partial sclerosis was observed in part of the periphery of the tumor (Figure 1(b)). Magnetic resonance imaging (MRI) showed a 4.5 × 3.5 cm mass penetrating into soft tissue as detected by CT (Figure 2). The mass had multiple cystic components with fluid-fluid levels indicative of different densities of fluid collections after the hemorrhage. Low signal intensity at the periphery of the multiple cysts was noted on both T1- and T2-weighted images, suggesting hemosiderin deposits or fibrous tissue. T2- and gadolinium-enhanced T1-weighted images depicted a high signal intensity rim superficial to the low-signal intensity rim. Gadolinium-enhanced T1-weighted images showed no enhancement of the cystic lesions, and moderate enhancement was observed in the solid lesions with iso signal intensity on T1-weighted images and high signal intensity on T2-weighted images. Partial infiltration into the surrounding bone tissue and soft tissue was observed on T2- and gadolinium-enhanced T1-weighted images. Fluorodeoxyglucose positron emission tomography (FDG PET/CT) depicted uptake of FDG in the tumor with SUVmax of 4.16, and a small lymph node swelling with SUV max of 2.8, which was suspected to be metastasis from the tumor or a reactive lymph node swelling (Figure 3). These imaging findings evoked the differential diagnosis of aneurysmal bone cysts and bone sarcomas with low-to high-grade malignant potential.

**Figure 4:**
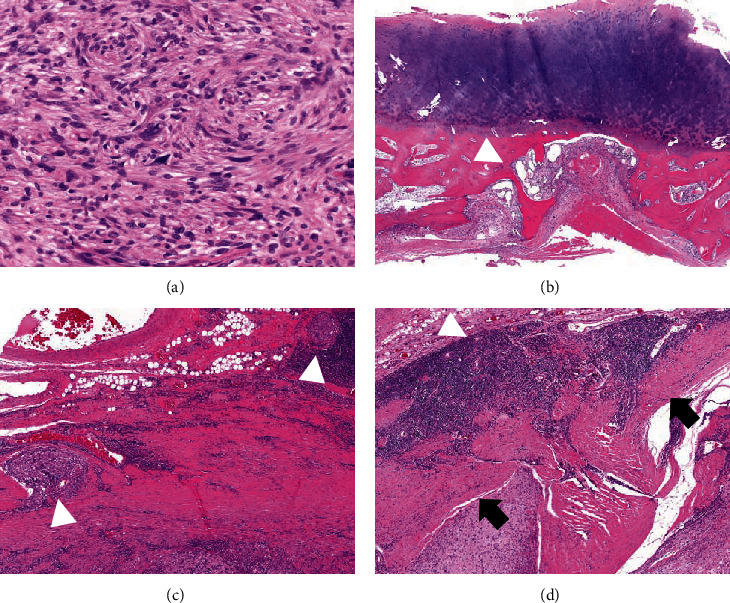
Hematoxylin and eosin staining of the tumor tissue. (a) Proliferation of spindle cells with high cellularity and atypical cells with bizarre nuclei are observed. (b) Tumor cells (white arrowhead) infiltrate the subchondral bone under the glenoid cartilage. (c) Tumor cells (white arrowhead) invading surrounding fibroadipose tissue, and (d) lymphoplasmacytic infiltration (white arrowhead) surrounding fibrous capsule (black arrow).

Histopathological examination of the CT-guided needle biopsy resulted in a diagnosis of spindle cell sarcoma (Figure 4). Immunohistochemically, the tumor cells expressed focal positivity for epithelial membrane antigen (EMA) and Bcl-2, and diffuse positivity for CD99, and were negative for desmin, CD34, AE1/AE3, and S-100. After a multidisciplinary conference, the patient received neoadjuvant chemotherapy with two courses of cisplatin and doxorubicin because of the possibility of a high-grade sarcoma. The size of the tumor did not change after chemotherapy, but cystic changes in the solid part of the tumor progressed (Figure 2(d)), and the accumulation of FDG at the axillary lymph node was diminished. Total scapulectomy and resection of the axillary lymph nodes were performed. The macroscopic appearance of the resected tumor was an ill-circumscribed lobulated mass with cystic changes. Microscopically, spindle cells with high cellularity and partially atypical cells with bizarre nuclei were observed (Figure 4). Immunohistochemical profile was similar to the needle biopsy, and the histopathological diagnosis of the resected specimen was also spindle cell sarcoma with the differential diagnosis of undifferentiated pleomorphic sarcoma and synovial sarcoma. Viable tumor cells were observed in most of the tumor, suggesting limited efficacy of chemotherapy, and no metastatic tumor cells were observed in the axillary lymph nodes. Focal invasion of the tumor cells into the surrounding bone and soft tissue was noted, and lymphoplasmacytic infiltration was observed around the fibrous capsule of the tumor (Figure 4). After resection of the tumor, the C-reactive protein level normalized, and the patient had no metastasis or recurrence 5 years after the surgery.

Because of the difficulty of histopathological diagnosis, targeted next-generation sequencing (NGS) was performed after informed consent was obtained. RNA was extracted from the paraffin-embedded tumor tissue. The quality of extracted RNA was checked, and cDNA synthesis and library preparation for junction capture were conducted using a Sureselect RNA capture kit (Agilent Technologies, Santa Clara, CA). Custom-made probes for our Todai OncoPanel (TOP) include 464 cancer-related fusion genes [12]. Massively parallel sequencing of the isolated fragments was performed using the NextSeq 500 platform (Illumina, San Diego, CA, USA). NGS analysis using TOP identified the *EWSR1-CREB1* fusion gene, resulting in the diagnosis of AFH in combination with histological findings.

## 3. Discussion

We report a case of AFH of the bone in an elderly patient. Unusual location and older age of onset made the diagnosis in this case difficult. However, comprehensive genetic testing, especially RNA-sequencing, was able to detect disease-specific fusion genes. In addition, imaging findings of multiple cystic lesions with fluid-fluid levels and paraneoplastic symptoms of elevated systemic inflammation could suggest this specific diagnosis regardless of the unusual location and age of onset.

Targeted RNA sequencing revealed a characteristic fusion gene of AFH, the *EWSR1-CREB1* fusion. A previous report has described that AFH can have three fusion genes: more than 90% of cases possess the *EWSR1-CREB1* fusion, less common is the *EWSR1-ATF1* fusion, and a few cases harbor the *FUS-ATF1* fusion [8, 9]. These fusion genes are not specific to AFH. Clear cell sarcoma, which is a highly malignant soft tissue sarcoma, possesses *EWSR1-CREB1* in 6% of cases and *EWSR1-ATF1* fusion in 94% of cases, as previously reported [8]. Therefore, the results of genetic testing should be interpreted along with the histopathological findings. In our case, a definite diagnosis was not achieved with the analysis of biopsy and resected specimen because the tumor was located at an unusual site, which was not soft tissue but bone, and the presentation was at an older age than the average age of AFH. However, targeted RNA sequences using paraffin-embedded specimens and original designed RNA panels, including genes related to bone and soft tissue tumors, could detect *EWSR1-CREB1* and help to arrive at the correct diagnosis.

The imaging findings of MRI in our case possess similar characteristics to AFH in the soft tissue. A limited case series and case reports have mentioned the characteristics of imaging features of AFH in soft tissue, and AFH is seen to have a combination of solid and multiple cystic lesions filled with blood products, occasionally representing fluid-fluid levels [10, 11]. Martinez et al. reported that AFH of soft tissue represents a double rim sign in 80% of cases through the analysis of six cases and a literature review [11]. The double rim sign is an imaging feature of the periphery of the tumor and the presence of a rim of high signal intensity (RHS) surrounding a rim of low signal intensity (RLS) on T2-weighted and gadolinium-enhanced images. The RLS reflects the pseudocapsule, which may contain fibrous tissue and hemosiderin deposition. In contrast, RHS represents the inflammatory layer of lymphocytes and plasma cells. In addition, they reported that partial tumor infiltration penetrating the two-layer rim was observed in 60% of cases. Consistent with this report of AFH of soft tissue, our case presented with multiple cystic lesions with fluid-fluid levels and some solid lesions. These solid lesions partially changed to cystic lesions after the two courses of chemotherapy. It is unclear whether this cystic change reflected a chemotherapeutic effect or not, and this change could indicate the nature of this disease because many viable cells were observed in the resected specimen. Furthermore, infiltration into the surrounding tissue, especially into the soft tissue, was also observed in our case. Previous reports of AFH of bone also reported sharing the imaging characteristics of AFH of soft tissue [13]. Therefore, these imaging features suggest the possibility of AFH even if the tumors are located at sites other than the soft tissue. As for the treatment strategy, six cases of AFH of bone have been reported, and all three cases treated with curettage had local recurrence [2]. This result is supported by the imaging findings of partial infiltration. Considering the infiltrative nature of AFH, AFH of the bone should be treated with wide resection, and curettage should be avoided.

AFH is known to cause local lymph node swelling. Ulaner et al. reported that seven out of 17 cases of primary AFH of soft tissue had local lymph node swelling [14]. Only one of these cases was a metastatic lesion, while the others were benign. Swelling of the axillary lymph node, which could be a regional lymph node of the scapula, was observed in our case, and the resected specimen revealed that the lesion was benign. This is the first case in which AFH of the bone also had a local lymph node swelling. Furthermore, patients with AFH rarely present with paraneoplastic syndromes, including weight loss, anemia, fever, night sweats, nausea, and vomiting [7]. These symptoms are associated with IL-6 and TNF-*α* released by the tumor, and this production is thought to be related to the *EWSR1-CREB1* fusion gene as a transcriptional factor. A retrospective review of our case, the existence of regional lymph node swelling, and elevation of C-reactive protein could have helped in the diagnosis of AFH.

In conclusion, we presented a case of AFH of bone with an uncommon location and age, and targeted RNA sequencing could support the histopathological diagnosis. Imaging findings were identical to those of AFH in soft tissues, paraneoplastic symptoms of elevated systemic inflammatory markers, and local lymph node swelling might have aided the histopathological diagnosis, which evokes the importance of Jaffe's triangle. Furthermore, in addition to clinical, radiological, and histological information, genetic information can help arrive at a correct diagnosis even for rare and uncommon tumors, such as AFH.

## Data Availability

The datasets used and/or analyzed in the current work are available from the corresponding author upon reasonable request. All data generated or analyzed during this study are included in this article.
